# A Federal Landscape Assessment and the Path Toward a Connected Society

**DOI:** 10.1093/geroni/igaf122.1044

**Published:** 2025-12-31

**Authors:** Andrew MacPherson

**Affiliations:** Healthsperien LLC, Washington, District of Columbia, United States

## Abstract

Public policy plays a critical role in shaping the environments, systems, and structures that influence social connection. Drawing upon the Foundation for Social Connection Action Network’s extensive advocacy experience, this discussion will provide attendees with a comprehensive understanding of the evolving political landscape, recent legislative advancements, and opportunities for bipartisan action to strengthen social engagement for older adults. Particular attention will be paid to the Older Americans Act (OAA), a cornerstone of aging policy that funds critical programs supporting social connection, and the Safeguarding Elderly Needs through Innovation and Occupational Resources (SENIOR) Act of 2024, which introduces new measures to address social isolation through workforce development and community-based initiatives. Attendees will also be introduced to the Action Network’s Policy Priorities for the 119th Congress - an evidence-based, cross-sector agenda designed to embed social connection at the heart of public policy.

